# Chronic Cyclodextrin Treatment of Murine Niemann-Pick C Disease Ameliorates Neuronal Cholesterol and Glycosphingolipid Storage and Disease Progression

**DOI:** 10.1371/journal.pone.0006951

**Published:** 2009-09-11

**Authors:** Cristin D. Davidson, Nafeeza F. Ali, Matthew C. Micsenyi, Gloria Stephney, Sophie Renault, Kostantin Dobrenis, Daniel S. Ory, Marie T. Vanier, Steven U. Walkley

**Affiliations:** 1 Dominick P. Purpura Department of Neuroscience, Rose F. Kennedy Center for Research in Mental Retardation and Human Development, Albert Einstein College of Medicine, Bronx, New York, United States of America; 2 Department of Medicine, Washington University School of Medicine, St. Louis, Missouri, United States of America; 3 Department of Cell Biology and Physiology, Washington University School of Medicine, St. Louis, Missouri, United States of America; 4 Institut National de la Sante et de la Recherche Medicale Unit 820, Laennec Medical School and Lyon-1 University, Lyon, France; University of Florida, United States of America

## Abstract

**Background:**

Niemann-Pick type C (NPC) disease is a fatal neurodegenerative disorder caused most commonly by a defect in the NPC1 protein and characterized by widespread intracellular accumulation of unesterified cholesterol and glycosphingolipids (GSLs). While current treatment therapies are limited, a few drugs tested in *Npc1^−/−^* mice have shown partial benefit. During a combination treatment trial using two such compounds, *N-*butyldeoxynojirimycin (*N*B-DNJ) and allopregnanolone, we noted increased lifespan for *Npc1^−/−^* mice receiving only 2-hydroxypropyl-β-cyclodextrin (CD), the vehicle for allopregnanolone. This finding suggested that administration of CD alone, but with greater frequency, might provide additional benefit.

**Methodology/Principal Findings:**

Administration of CD to *Npc1^−/−^* mice beginning at either P7 or P21 and continuing every other day delayed clinical onset, reduced intraneuronal cholesterol and GSL storage as well as free sphingosine accumulation, reduced markers of neurodegeneration, and led to longer survival than any previous treatment regime. We reasoned that other lysosomal diseases characterized by cholesterol and GSL accumulation, including NPC disease due to NPC2 deficiency, GM1 gangliosidosis and mucopolysaccharidosis (MPS) type IIIA, might likewise benefit from CD treatment. Treated *Npc2^−/−^* mice showed benefits similar to NPC1 disease, however, mice with GM1 gangliosidosis or MPS IIIA failed to show reduction in storage.

**Conclusions/Significance:**

Treatment with CD delayed clinical disease onset, reduced intraneuronal storage and secondary markers of neurodegeneration, and significantly increased lifespan of both *Npc1^−/−^* and *Npc2^−/−^* mice. In contrast, CD failed to ameliorate cholesterol or glycosphingolipid storage in GM1 gangliosidosis and MPS IIIA disease. Understanding the mechanism(s) by which CD leads to reduced neuronal storage may provide important new opportunities for treatment of NPC and related neurodegenerative diseases characterized by cholesterol dyshomeostasis.

## Introduction

Niemann-Pick type C (NPC) disease is an autosomal recessive neurodegenerative disorder characterized by accumulation of unesterified cholesterol and glycosphingolipids (GSLs), such as GM2 and GM3 ganglioside [1], [2]. Patients with this fatal disease develop an ataxic gait and motor dysfunction, typically preceded by vertical gaze palsy and organomegaly, and later accompanied by seizures and dementia. On a cellular level, neurons exhibit complex disease-related morphological alterations including formation of meganeurites, ectopic dendrites, and axonal spheroids [3]. NPC disease is also characterized by neurodegeneration, including the presence of intracellular protein aggregates in the form of neurofibrillary tangles, and a well-defined patterned loss of cerebellar Purkinje cells [4]. Defects in a transmembrane protein, NPC1, account for approximately 95% of cases with the remainder involving a soluble protein, NPC2 [2]. Both proteins have been shown to bind cholesterol [5]–[8] and are found in the late endosomal/lysosomal (LE/LY) system. Here they are thought to function cooperatively in facilitating egress of cholesterol from LE/LY to other sites in the cell [9], [10]. Cholesterol sequestration in NPC disease is generally believed to be followed secondarily by GSL accumulation, but restricting synthesis of complex gangliosides has been shown to reduce cholesterol storage in most neurons [11], [12].

Therapeutic options for NPC disease are quite limited. Substrate reduction therapy (SRT) utilizes drugs that reduce the synthesis of metabolic precursors or products which themselves are known to accumulate in storage diseases [13]. In 2001, Zervas and colleagues demonstrated that daily administration of an inhibitor of GSL synthesis, *N*-butyldeoxynojirimycin (*N*B-DNJ or miglustat) to *Npc1^−/−^* mice resulted in a reduction in accumulation of GSLs, a delay in onset of clinical signs, and a 30% increase in lifespan [14]. More recently, miglustat has also been shown in a randomized controlled study to stabilize or improve clinical markers of NPC disease in patients treated for 12 months [15] and in January of 2009, miglustat (Zavesca®) was approved by the European Medicines Agency for use in treatment of neurological symptoms in patients with NPC disease. A second therapeutic agent tested in *Npc1^−/−^* mice was allopregnanolone, a neurosteroid shown deficient in the central nervous system (CNS) of *Npc1^−/−^* mice [16]. Administration of allopregnanolone solubilized in 2-hydroxypropyl-β-cyclodextrin (CD) to *Npc1^−/−^* mice at postnatal day 7 (P7) was reported to be beneficial, with treated mice exhibiting delayed clinical onset, extended life span, and reduced ganglioside accumulation. This therapeutic approach has been referred to as by-product replacement therapy or BRT, since the strategy was thought to replace missing or deficient products needed for normal cellular functioning [17]. Based on these positive results, we reasoned that a combination therapy utilizing both miglustat and allopregnanolone might work synergistically to ameliorate NPC1 disease. While this combination therapy did prove additive, our studies revealed that the vehicle, CD, also provided significant benefit. This finding coincided with published observations that single dose CD increased the lifespan of *Npc1^−/−^* mice (18). Additional studies reported here further establish that CD treatment alone dramatically ameliorates NPC disease and questions the efficacy of allopregnanolone administered without CD. A recent publication investigating the effects of a single injection of CD with or without allopregnanolone in NPC1 disease reported no additional benefit of allopregnanolone in reducing cholesterol accumulation in the liver and brain as well as a reduction in markers of neurodegeneration, but did reconfirm the increase in lifespan of CD-treated *Npc1^−/−^* mice. [19]. Our current report further demonstrates that sequestration of GSLs, sphingosine, and cholesterol is significantly reduced in neurons of CD-treated *Npc1^−/−^* mice and that chronic treatment with CD leads to the most significant amelioration of NPC disease in the murine model seen to date.

## Results

### Combination SRT and BRT therapy in Npc1^−/−^ mice provides synergistic effect

**Figure 1:**
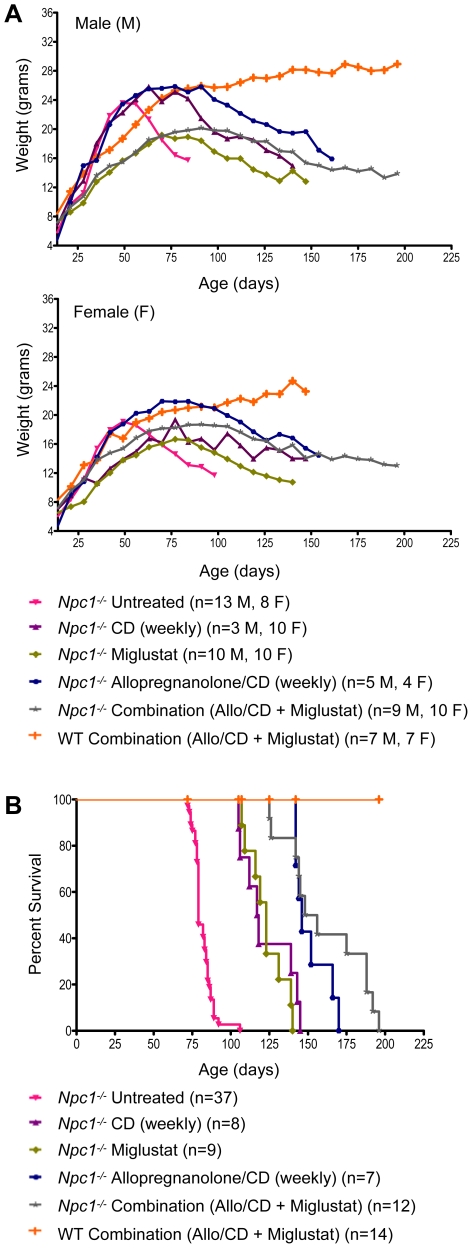
Combination treatment using *N*B-DNJ and allopregnanolone/CD in *Npc1^−/−^* mice. (A) Average weight over time for each treatment group shown for males and females separately. (B) Survival of each treatment group. Median survival of *Npc1^−/−^* mice: no treatment, 79 days; CD (weekly), 118 days; Miglustat (daily), 123 days; Allopregnanolone/CD (weekly), 146 days; Combination therapy (Miglustat + Allopregnanolone/CD), 152 days.

**Figure 2:**
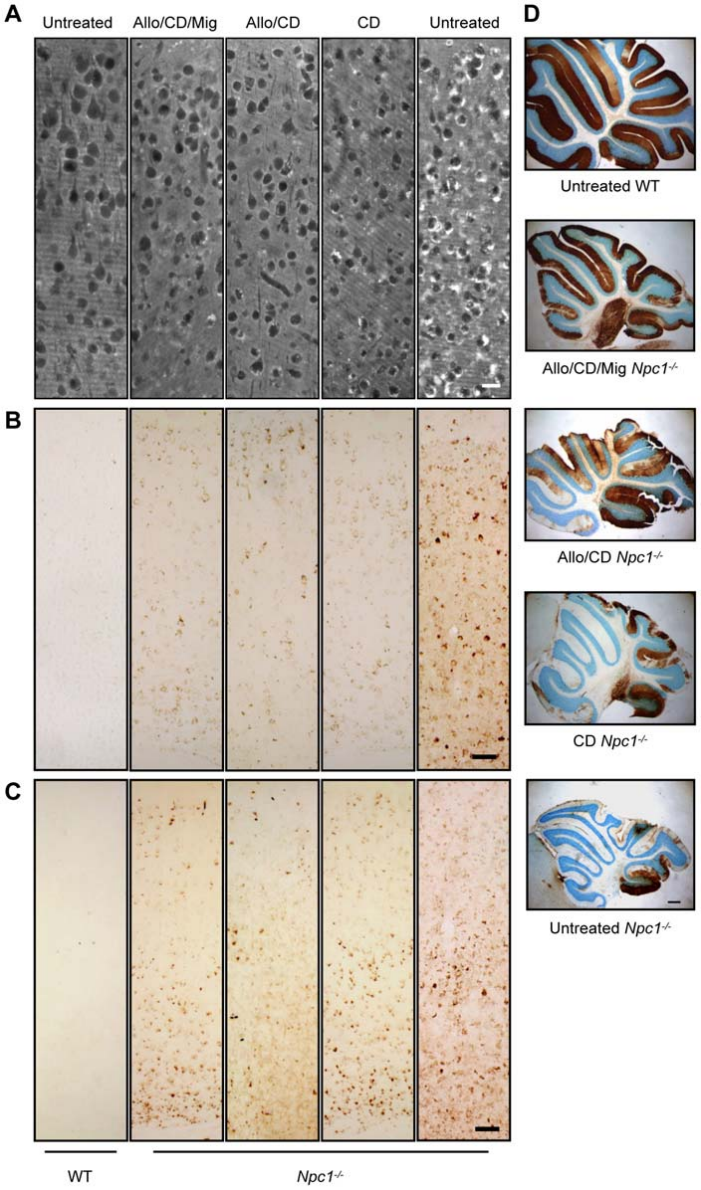
Cholesterol and ganglioside immunohistochemistry (IHC) of *Npc1^−/−^* and WT mice in the combination treatment study. (A) Filipin labeling of unesterified cholesterol (seen as white areas in image) in the neocortex of age-matched untreated and treated *Npc1^−/−^* and WT mice (all mice between 75 and 81 days of age) revealed less cholesterol accumulation of treated *Npc1^−/−^* mice (second, third, and fourth panels) when compared to control *Npc1^−/−^* mice (fifth panel). WT mice do not exhibit cholesterol accumulation (first panel). Each panel here and in (B) and (C) shows layers II (top) through VI (bottom) of the cerebral cortex. (B) IHC of GM2 ganglioside (visualized as brown punctae within cells) was also characterized by reduced GM2 storage in all treated *Npc1^−/−^* mice. (C) IHC of GM3 ganglioside (again seen as brown punctae within cells) showed results similar to GM2. (D) Treated *Npc1^−/−^* mice had more remaining Purkinje cells (brown areas in cerebellar images) than did untreated *Npc1^−/−^* mice; however, treated mice still had Purkinje cell loss when compared to WT mice. Anti-calbindin antibody labels Purkinje cell bodies and dendritic arbors, while the Nissl counterstain (purple) labels all neuronal cell bodies. Images taken at 20X (A), 10X (B, C), and 2X (D); scale bars 20 µm (A), 50 µm (B, C), and 400 µm (D).

*Npc1^−/−^* mice treated with a combination therapy of *N*B-DNJ (administered daily starting at P10) and allopregnanolone/CD (administered weekly starting at P7) showed a 2 week delay in onset of clinical signs (ataxic gait, tremor) when compared to untreated (given no injections) or vehicle (saline or CD) treated *Npc1^−/−^* mice. Untreated *Npc1^−/−^* mice began a precipitous weight loss beginning at 6 weeks of age, while combination-treated *Npc1^−/−^* mice gradually lost weight starting at 14 weeks (Fig. 1A). Combination-treated *Npc1^−/−^* mice lived significantly longer than did untreated or vehicle-injected (saline or CD) *Npc1^−/−^* mice (Fig. 1B). We noted, however, that *Npc1^−/−^* mice receiving CD alone also lived significantly longer than untreated *Npc1^−/−^* mice (median age: untreated *Npc1^−/−^* mice: 79 days; CD-treated *Npc1^−/−^* mice: 118 days; p<0.0001). Correspondingly, weight loss in CD-injected *Npc1^−/−^* mice was delayed by approximately 3 weeks. Analysis of cholesterol accumulation by filipin labeling revealed a decrease in cholesterol storage in neocortical neurons of combination and CD-treated *Npc1^−/−^* mice compared to untreated *Npc1^−/−^* mice (Fig. 2A). Like cholesterol, GM2 and GM3 gangliosides are well characterized storage components of NPC disease, but their levels in wild-type (WT) brain are negligible. Immunohistochemical (IHC) analysis of GM2 and GM3 gangliosides in the neocortex of *Npc1^−/−^* mice yielded similar results in terms of cholesterol storage, with combination and CD-treated mice exhibiting less ganglioside accumulation than untreated mice (Fig. 2B, C). Comparison of cerebellum in age-matched untreated and combination-treated *Npc1^−/−^* mice revealed a striking rescue of Purkinje cells in treated animals as evidenced by calbindin labeling (Fig. 2D). While surviving Purkinje cells were confined largely to lobule X in untreated *Npc1^−/−^* mice, those receiving the combination drug treatment still had Purkinje cells present in all lobules. GM2 accumulation was present mainly in the granular cell layer of *Npc1^−/−^* mice, but combination-treated *Npc1^−/−^* animals had less GM2 staining than *Npc1^−/−^* controls (untreated and saline; data not shown).

### Short-term administration of CD alone reduces storage in Npc1^−/−^ mice

**Figure 3:**
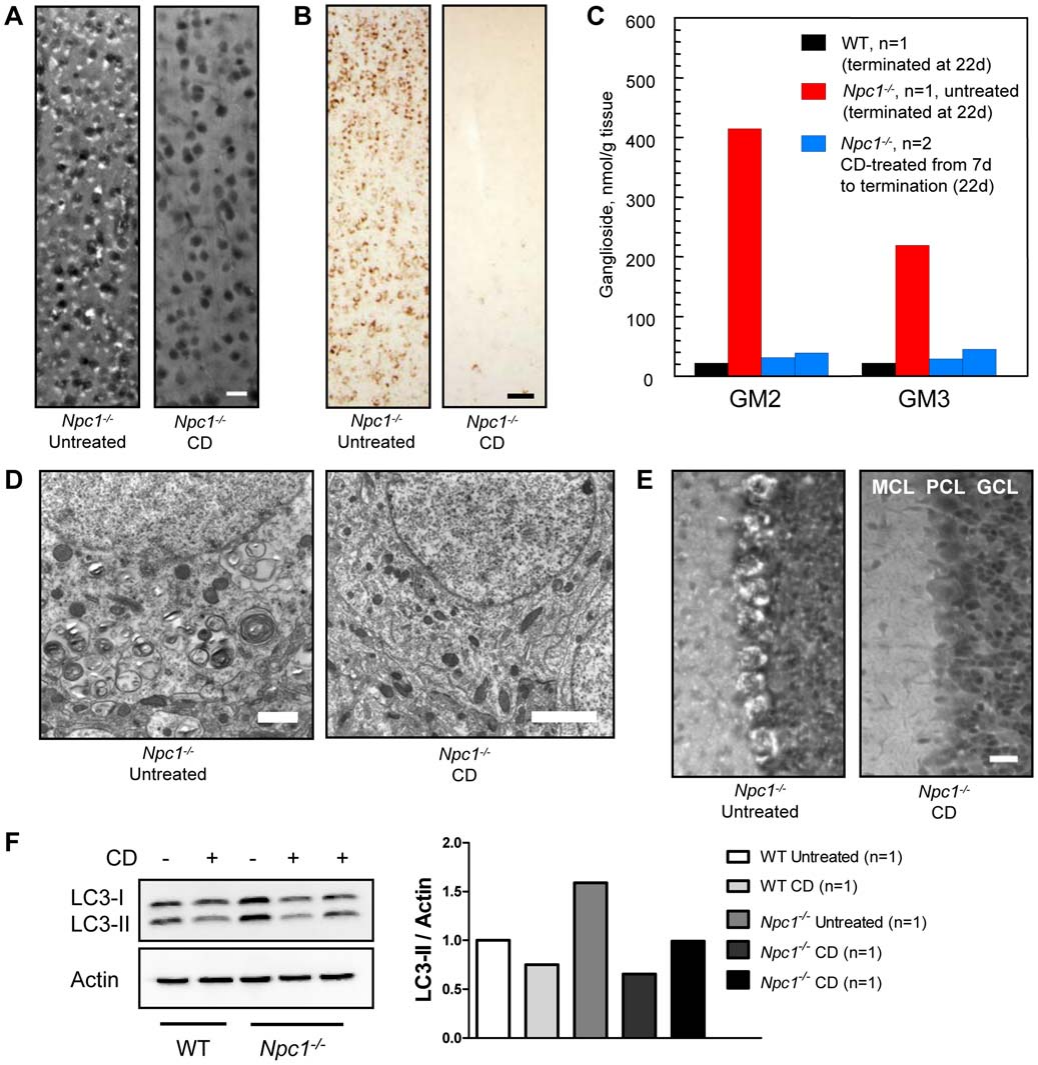
Short term (2 week) CD study in *Npc1^−/−^* mice. (A) Filipin labeling of unesterified cholesterol in the neocortex of untreated and CD-treated *Npc1^−/−^* mice revealed dramatically less cholesterol accumulation in CD-treated mice at 22 days of age. Mice were administered SC injections of CD every other day for 2 weeks starting at P7. (B) IHC of untreated and CD-treated *Npc1^−/−^* mice also revealed less GM2 storage present in CD-treated mice (similar finding for GM3, not shown). (C) Biochemical analysis of ganglioside levels further corroborated the reduction in GM2 and GM3 seen with IHC analysis. (D) Ultrastructural analysis of neocortical neurons in *Npc1^−/−^* untreated and CD-treated mice showed remarkably normal neuronal morphology in CD-treated mice. (E) Filipin labeling of unesterified cholesterol in the cerebellum of untreated and CD-treated *Npc1^−/−^* mice indicated little to no cholesterol accumulation present within Purkinje cells of CD-treated mice; cerebellar layers: molecular cell layer (MCL), Purkinje cell layer (PCL), and granular cell layer (GCL). (F) Western blot analysis of LC3-II, an autophagosome marker, revealed less LC3-II present in CD-treated *Npc1^−/−^* as compared to untreated *Npc1^−/−^* mice. Images taken at 20X (A, E) and 10X (B); scale bars 20 µm (A, E), 50 µm (B), 1 µm (D).

The discovery that CD alone (without allopregnanolone) reduced intraneuronal storage and increased longevity of *Npc1^−/−^* mice led us to perform a series of studies to address its possible role as a therapeutic agent in and of itself. *Npc1^−/−^* mice were treated beginning at P7 with injections of CD every other day for 2 weeks. Route of CD administration was either subcutaneous (SC) or intraperitoneal (IP) and while both had a similar outcome, SC administration seemed to be slightly more efficacious (Fig. S1). At 2 weeks of age, mice were terminated to evaluate cholesterol and GSL storage. Results showed little to no accumulation of cholesterol in neurons of the cerebral cortex in 22-day old treated *Npc1^−/−^* mice, whereas age-matched untreated *Npc1^−/−^* mice exhibited considerable filipin-labeling (Fig. 3A). CD treatment also diminished accumulation of gangliosides, such that GM2 and GM3 storage in the neocortex of treated *Npc1^−/−^* mice was nearly absent compared to readily detectable storage in untreated *Npc1^−/−^* mice (Fig. 3B; GM3 not shown). A parallel quantitative biochemical study of gangliosides in cerebrum of these mice revealed strikingly different patterns in the untreated and treated *Npc1^−/−^* animals, with a near normalization to WT levels in the concentrations of both GM2 and GM3 after CD treatment (Fig. 3C). At this age, GM2 has already reached its near maximal level in the untreated mutants, while GM3 is at about 50% of its maximum (unpublished data, MT Vanier). Ultrastructural analysis revealed that cortical neurons of a 22-day old CD-treated *Npc1^−/−^* mouse had little to no evidence of polymembranous cytoplasmic bodies (PCBs) characteristic of NPC disease (Fig. 3D), a finding consistent with the lack of detectable cholesterol and ganglioside storage.

Analysis of cerebellar cortex from 22-day old untreated *Npc1^−/−^* mice revealed that nearly every Purkinje cell, as well as presumptive neurons in both the granule and molecular cell layers, showed evidence of cholesterol accumulation by filipin labeling (Fig. 3E). However, most Purkinje cells in age-matched CD-treated *Npc1^−/−^* mice lacked cholesterol storage and only a small number of filipin-positive neurons were present in the granule cell layer. IHC analysis of GM2 in the cerebellum of untreated *Npc1^−/−^* mice revealed prominent accumulation throughout the granule cell layer and occasional storage in the molecular cell layer, while age-matched CD-treated *Npc1^−/−^* mice exhibited less accumulation in both the granule and molecular cell layers (data not shown). Levels of the autophagosome marker LC3-II were also affected by CD treatment, as evidenced by western blot analysis of the cerebellum. Increased levels of LC3-II have previously been shown in the CNS of *Npc1^−/−^* mice suggesting alterations in the degradative mechanism known as macroautophagy [20], [21]. Our results confirmed this increase in LC3-II and additionally, showed that levels in CD-treated *Npc1^−/−^* mice were normalized to those seen in WT controls (Fig. 3F).

These findings indicating that CD significantly limits cholesterol and ganglioside storage in neurons of young *Npc1^−/−^* mice led us to carry out two additional studies, one examining the ability of allopregnanolone alone to ameliorate disease progression and the other to determine the efficacy of long-term CD therapy.

### Allopregnanolone without CD does not appear beneficial

**Figure 4:**
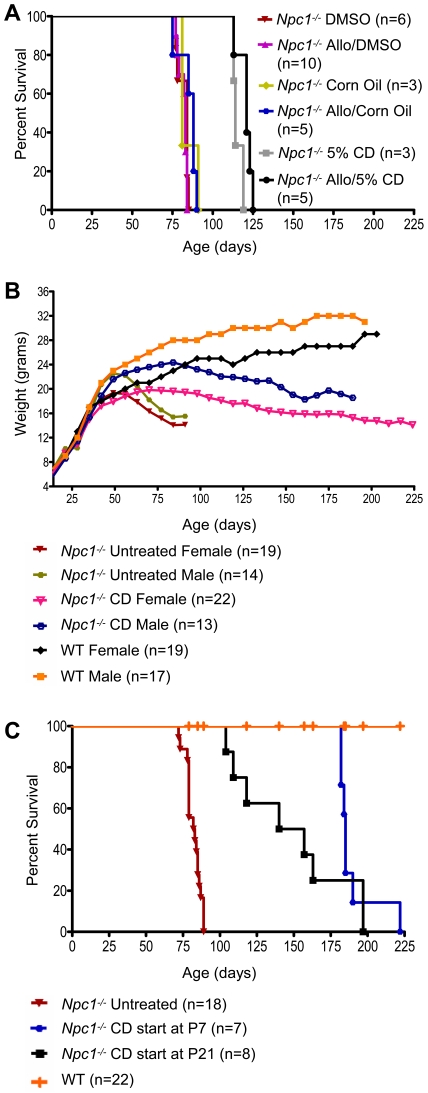
Chronic allopregnanolone and chronic CD treatment studies in *Npc1^−/−^* mice. (A) Survival of untreated and allopregnanolone-treated *Npc1^−/−^* mice using different vehicles for allopregnanolone. Median survival of *Npc1^−/−^* mice: DMSO, 84 days; Allo/DMSO, 83 days; Corn Oil, 81 days; Allo/Corn Oil, 88 days; 5% CD, 114 days; Allo/5% CD, 121 days. (B) Average weight over time for untreated and chronically CD-treated *Npc1^−/−^* and WT mice. Weights of untreated and CD-treated WTs were averaged for each gender as there was no significant difference between treatments (p<0.8125). (C) Survival of untreated and CD-treated *Npc1^−/−^* and WT mice showing effects of different start times. Median survival of *Npc1^−/−^* mice: no treatment, 83 days; CD (every other day, start at P7), 185 days; CD (every other day, start at P21), 149 days. Treatment initiated at P7 appeared more efficacious, although lifespan was not significantly longer when compared to treatment initiated at P21 (p<0.1870).

The initial combination study suggested not only a beneficial effect of CD, but also a possible small additional benefit of allopregnanolone with CD (Fig. 1A; median age: CD-treated *Npc1^−/−^* mice: 118 days; Allopregnanolone/CD-treated *Npc1^−/−^* mice: 146 days; p<0.0038). To determine whether there was any beneficial impact of allopregnanolone alone on *Npc1^−/−^* mice, we administered allopregnanolone using vehicles other than CD (dimethyl sulfoxide [DMSO] or corn oil) or a reduced concentration of CD (5% CD solution). Mice were given weekly injections following the same protocol used in the combination study, with the exception of allopregnanolone/corn oil (total of three injections given at P7, P14, and P21). Onset of ataxic gait occurred in all treated and untreated *Npc1^−/−^* mice at 6–7 weeks of age and there was no increase in lifespan of the allopregnanolone-treated *Npc1^−/−^* mice (vehicle: corn oil or DMSO) compared to vehicle injected *Npc1^−/−^* controls. *Npc1^−/−^* mice treated with allopregnanolone solubilized in 5% CD did live significantly longer than mice receiving the other allopregnanolone solutions or corn oil and DMSO vehicles, but so too did the control animals receiving only 5% CD (Fig. 4A). Analysis of cholesterol and gangliosides did not demonstrate detectable storage reductions between the allopregnanolone-treated *Npc1^−/−^* mice versus their respective vehicle-injected controls. Collectively, these results suggest that the beneficial effects observed in the combination trial for allopregnanolone-treated *Npc1^−/−^* mice are due largely to the vehicle, CD, and that allopregnanolone may provide only a small additional benefit when administered in 20% CD.

### Chronic CD injections ameliorate NPC1 disease in mice

Using the same treatment regimen described earlier (injections given every other day), *Npc1^−/−^* and WT mice were administered CD beginning at P7 and continuing to end-stage disease, when affected mice showed a hunched posture, severe gait disturbance, and/or weight loss greater than 30% of peak weight. Behaviorally, onset of ataxia in untreated *Npc1^−/−^* mice occurred between 6 and 7 weeks of age, while CD treatment delayed onset by 3 weeks, such that treated *Npc1^−/−^* mice became ataxic between 9 and 10 weeks of age. Furthermore, abrupt weight loss in untreated *Npc1^−/−^* mice began to occur around 7 weeks of age, while treated *Npc1^−/−^* mice exhibited a gradual decline in weight beginning at 10 to 13 weeks of age (Fig. 4B). Every-other-day treated *Npc1^−/−^* mice on average lived longer than those on the original combination therapy, though the difference was not significant (Fig. 1B and 4C; median age: combination-treated *Npc1^−/−^* mice: 152 days; CD-treated *Npc1^−/−^* mice began at P7: 185 days; p<0.2138).

**Figure 5:**
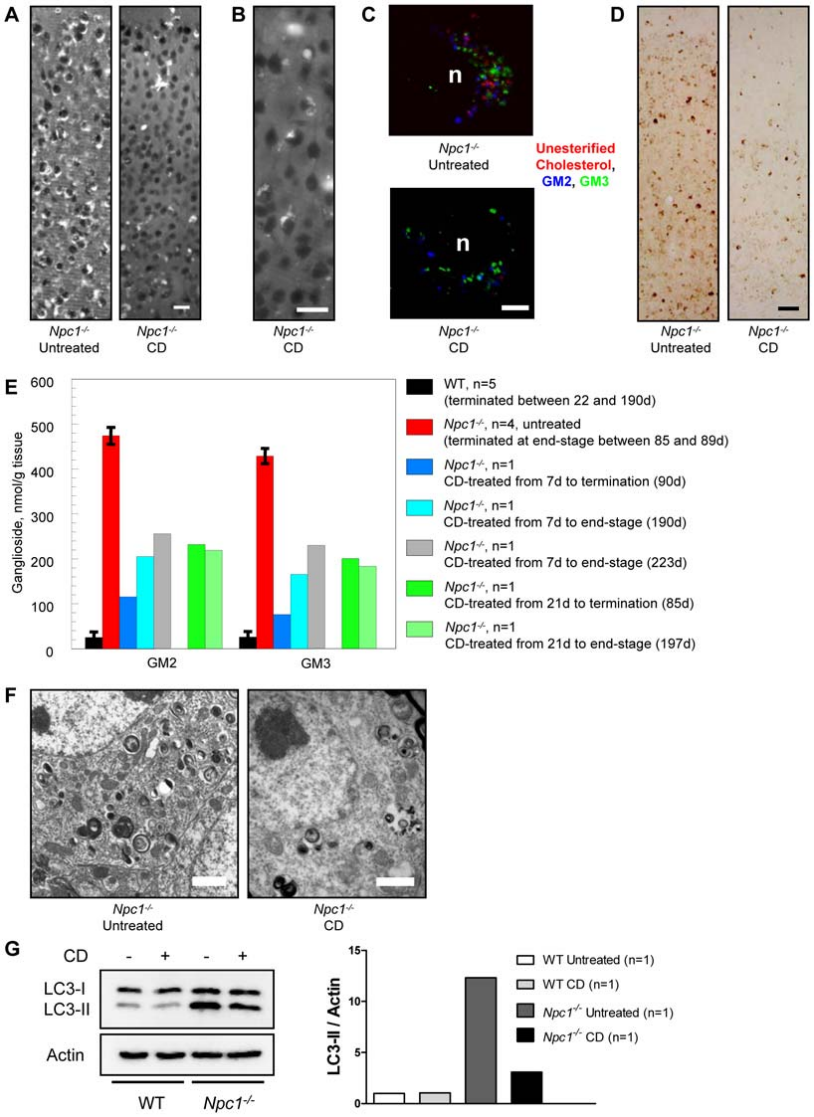
Chronic CD treatment study in *Npc1^−/−^* mice. (A) Filipin labeling of unesterified cholesterol in the neocortex of untreated (end-stage, 78 days old) and CD-treated (start at P21; end-stage, 197 days old) *Npc1^−/−^* mice showed reduced cholesterol accumulation in a CD-treated mouse. (B) Higher magnification of neocortex in same CD-treated *Npc1^−/−^* animal as previous panel, showed presence of neurons with cholesterol accumulation while neighboring cells lacked this storage. (C) Confocal microscopy further revealed that gangliosides and cholesterol appeared to always co-sequester within neurons in an untreated *Npc1^−/−^* mouse (end-stage, 78 days old; upper panel). However, some neocortical neurons in a CD-treated *Npc1^−/−^* mouse (start at P7; end-stage, 182 days old; lower panel) had little to no detectable cholesterol accumulation, yet still exhibited ganglioside storage. Cholesterol (red, visualized with BC Theta), GM2 (blue), and GM3 (green); n denotes nucleus of single neuron shown in each image. (D) IHC of untreated and CD-treated *Npc1^−/−^* mice (same mice as A), revealed less GM2 storage (also GM3, not shown) in the neocortex of a CD-treated mouse. (E) Biochemical analysis of ganglioside levels further corroborated the reduction in GM2 and GM3 seen with IHC. Data from WT and untreated mutant mice represent mean ± SD. (F) Ultrastructural analysis of neocortical neurons in untreated and CD-treated *Npc1^−/−^* mice (same mice as C) revealed presence of PCBs in both groups, but CD-treated mice appeared to have fewer of these storage bodies. (G) Western blot analysis of LC3-II in 85 day old untreated and CD-treated (start at P21) *Npc1^−/−^* and WT mice revealed a reduction in LC3-II levels in the CD-treated *Npc1^−/−^* mouse. Images taken at 20X (A), 40X (B), 63X (C), and 10X (D); scale bars 20 µm (A, B), 2 µm (C), 50 µm (D), 1 µm (F).

Filipin labeling of neocortical neurons in CD-treated *Npc1^−/−^* mice at end-stage revealed less cholesterol storage compared to their untreated counterparts, in spite of their significant age differences (Fig. 5A). There was a reduction in both the number of neurons that showed cholesterol storage and the amount of cholesterol in individual neurons, some of which appeared to have little or no accumulation (Fig. 5B). Confocal analysis of treated *Npc1^−/−^* mouse brain showed that many cholesterol negative neocortical neurons still exhibited ganglioside accumulation (Fig. 5C).

IHC staining of both GM2 and GM3 gangliosides revealed less accumulation in CD-treated *Npc1^−/−^* mice compared to age-matched *Npc1^−/−^* controls (Fig. 5D, GM3 not shown). Biochemical analysis of gangliosides (Fig. 5E) corroborated and extended this finding. In the cerebrum of CD-treated *Npc1^−/−^* mice, the increase in GM2 and GM3 levels, while not significant at 22 days (Fig. 3C), remained very moderate at 90 days (115 and 76 nmol/g for GM2 and GM3, respectively, compared with 474±19 and 429±27 nmol/g in age-matched, end-stage untreated *Npc1^−/−^* mice; mean ± SD). The two treated mice at end-stage (190 and 223 days) still showed concentrations of GM2 and GM3 gangliosides approximately half of those in end-stage untreated *Npc1^−/−^* mice. Neuro-inflammation is also an important component of many lysosomal diseases [22], and upon IHC staining of CD68, a CNS inflammatory marker, we noted reduced labeling in brains of end-stage CD-treated *Npc1^−/−^* mice compared to end-stage untreated *Npc1^−/−^* mice (data not shown). Ultrastructurally, although neurons of CD-treated *Npc1^−/−^* mice often had PCBs, they appeared to be less abundant than in untreated *Npc1^−/−^* mice (Fig. 5F).

Cholesterol storage was observed in all Purkinje cells of the cerebellum in treated and untreated *Npc1^−/−^* mice. At end-stage, remaining Purkinje cells were observed almost exclusively in lobule X, whereas in treated *Npc1^−/−^* mice (age-matched to end-stage untreated *Npc1^−/−^* mice), rescued Purkinje cells were routinely observed in other lobules. IHC analysis of GM2 ganglioside revealed prominent accumulation throughout the granule cell layer and occasional storage within the molecular cell layer of end-stage untreated *Npc1^−/−^* mice cerebella. CD-treated *Npc1^−/−^* mice exhibited this same pattern but with reduced GM2 accumulation (data not shown).

As in the short term study, biochemical analysis of LC3-II in the cerebellum revealed that CD-treated *Npc1^−/−^* mice had levels similar to WT, while untreated *Npc1^−/−^* mice showed an increase in LC3-II levels compared to WT (Fig. 5G).

In addition to brain analyses, we examined peripheral tissues including kidney, liver, and lung for changes induced by treatment with CD. Filipin analysis of kidneys from the chronic CD treatment study revealed no obvious differences between treated and untreated *Npc1^−/−^* mice, even though CD is thought to be excreted mainly intact in urine [23] (Fig. S2A). H&E staining of kidney also revealed no obvious changes with CD treatment in either *Npc1^−/−^* or WT mice (Fig. S3A). Cholesterol labeling of the liver of untreated *Npc1^−/−^* mice occurred primarily in hepatocytes, with treated mice showing a shift in cholesterol sequestration to apparent Kupffer cells, a change also observed when comparing WT untreated and treated mice (Fig. S2B). Large, lipid-laden hepatocytes were seen in the untreated *Npc1^−/−^* liver tissue but were largely undetectable in CD-treated *Npc1^−/−^* liver as visualized with H&E staining (Fig. S3B). A parallel biochemical study of liver lipids revealed a near normalization of the levels of unesterified cholesterol and sphingomyelin, as well as of bis(monoacylglycero)phosphate (BMP) and neutral glycolipids (glucosylceramide and lactosylceramide) in chronic CD-treated *Npc1^−/−^* mice (Fig. S4). Filipin analysis of lung revealed cholesterol laden cells in both untreated and CD-treated age-matched *Npc1^−/−^* mice (87 and 90 days, respectively), with a seemingly greater number of cholesterol positive cells present in the CD-treated animal. Lung from a CD-treated *Npc1^−/−^* mouse at end-stage disease (223 days) had a consolidated appearance with more lipid laden cells and cholesterol deposits throughout the tissue than either of the previous *Npc1^−/−^* mice (Fig. S2C). While H&E staining revealed the occasional presence of macrophages in untreated *Npc1^−/−^* lung, CD-treated *Npc1^−/−^* mouse lung showed more macrophages present and also increasing macrophage infiltration the longer CD-treatment was continued (Fig. S3C). Similar lipid-laden macrophages were not evident in either CD-treated or untreated WT lung.

In addition to starting CD treatment at 7 days of age, some *Npc1^−/−^* mice were treated shortly after weaning (P21–P25; post-weaning treated). Even with this two week delay, benefits similar to those seen in *Npc1^−/−^* mice treated beginning at P7 were still evident (Fig. 4C). Onset of clinical signs in *Npc1^−/−^* mice in which CD treatment was started post-weaning was delayed and longevity increased, although these mice did show greater variability than mice started at P7. Even though the median lifespan of these *Npc1^−/−^* mice treated post-weaning was less than that of mice started on treatment at P7, there was no significant difference (median age: CD-treated *Npc1^−/−^* mice starting post-weaning: 149 days; CD-treated *Npc1^−/−^* mice starting at P7: 185 days; p<0.1870). Cholesterol and ganglioside analysis of post-weaning treated *Npc1^−/−^* mice revealed reduction in storage compared to untreated *Npc1^−/−^* controls. This finding was supported by biochemical analysis of gangliosides in mice treated from P21 (Fig. 5E). Post-weaning treatment rescued Purkinje cells, although they still showed cholesterol accumulation similar to the *Npc1^−/−^* mice started on treatment at P7. Overall comparison of post-weaning treatment to treatment initiated at P7 suggests that although the latter seems slightly more effective in ameliorating NPC disease, initiating treatment at later dates still provides significant benefit to *Npc1^−/−^* mice.

### Chronic treatment of Npc2^−/−^ mice with CD ameliorates storage

**Figure 6:**
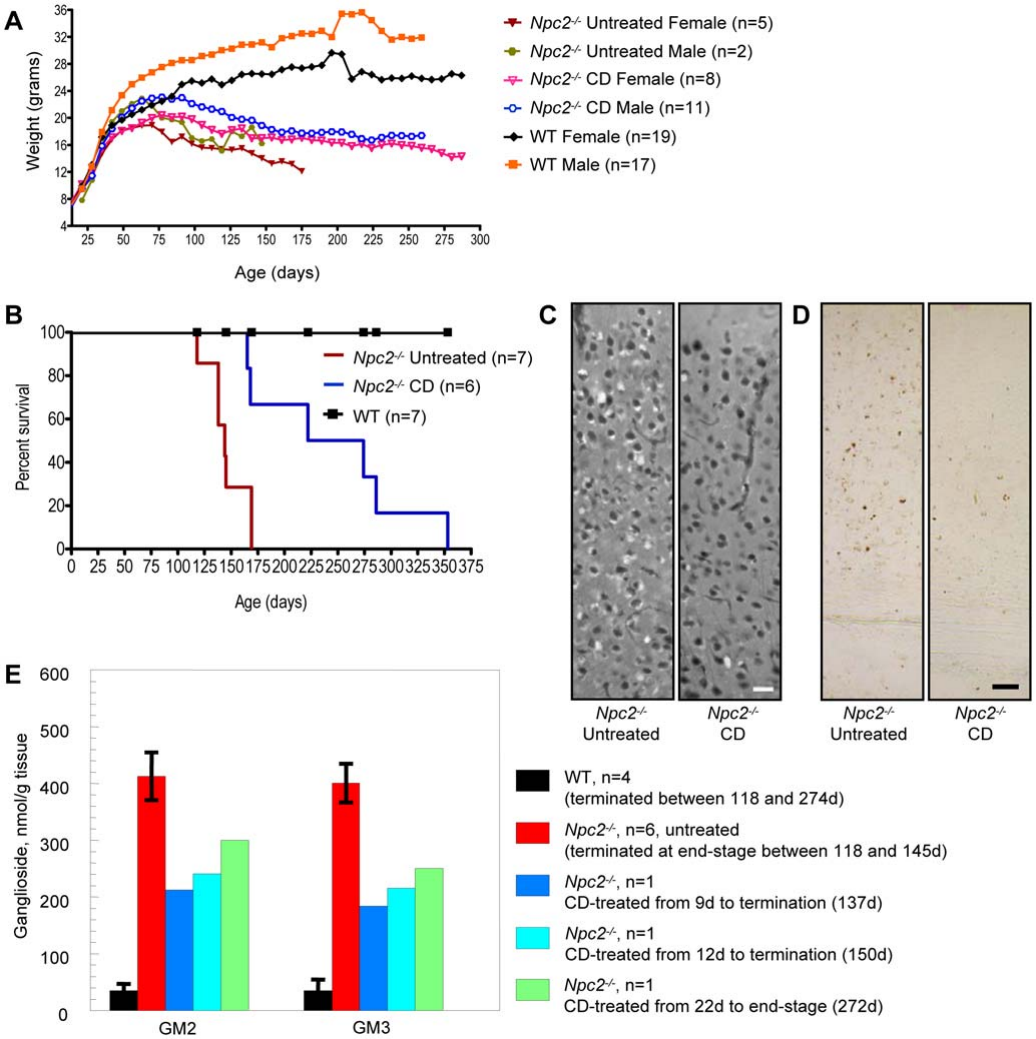
Chronic CD treatment study in *Npc2^−/−^* mice. (A) Average weight over time for untreated and CD-treated *Npc2^−/−^* and WT mice. Weights of untreated and CD-treated WTs were averaged for each gender as there was no significant difference between treatments (p<0.8125). (B) Survival of untreated and CD-treated *Npc2^−/−^* and WT mice. Median survival of *Npc2^−/−^* mice: no treatment, 144 days; CD (every other day), 248 days. CD-treated *Npc2^−/−^* mice lived significantly longer than untreated *Npc2^−/−^* mice (p<0.0108). (C) Filipin labeling of unesterified cholesterol in neocortex of untreated (end-stage, 145 days old) and CD-treated (start at P9; 150 days old) *Npc2^−/−^* mice revealed less cholesterol accumulation in the CD-treated mouse. (D) IHC of the same untreated and CD-treated *Npc2^−/−^* mice showed reduced GM2 labeling in the neocortex of the CD-treated mouse (also for GM3, not shown). (E) Biochemical analysis of ganglioside levels confirmed the reduction in GM2 and GM3 storage seen with IHC. Data from WT and mutant mice represent mean ± SD. Images taken at 20X (C) and 10X (D); scale bars 20 µm (C) and 50 µm (D).

Positive results from the CD treatment study in NPC1 disease led us to hypothesize that *Npc2^−/−^* mice might also benefit from treatment, as NPC2 disease is similarly characterized by cholesterol and GSL accumulation and the two NPC proteins may function in the same metabolic pathway [9], [10]. *Npc2^−/−^* and WT mice were administered CD following the same protocol used for *Npc1^−/−^* mice receiving chronic, every other day treatment, albeit at slightly different start dates. Onset of ataxic gait was delayed from 8 weeks of age in untreated *Npc2^−/−^* mice to approximately 10 weeks of age in treated *Npc2^−/−^* mice. Weight loss in untreated *Npc2^−/−^* mice began at approximately 10 weeks of age, while treated *Npc2^−/−^* mice did not show weight loss until 13 to 14 weeks of age (Fig. 6A). The average lifespan of an untreated *Npc2^−/−^* mouse is approximately 21 weeks of age and treatment with CD increased longevity by 11 weeks, such that on average, end-stage occurred at 32 weeks (Fig. 6B).

Filipin labeling of neocortical neurons in treated *Npc2^−/−^* mice showed reduced accumulation of unesterified cholesterol and IHC analysis of gangliosides revealed less GM2 and GM3 storage (Fig. 6C, D; GM3 not shown). By biochemical quantification (Fig. 6E), when treatment was initiated between P9 and P22, GM2 and GM3 gangliosides levels studied in mice at 137–150 days of age were reduced to about half of those in the untreated mice (212 and 241 nmol/g for GM2, 184 and 216 for GM3 in CD-treated *Npc2^−/−^* mice versus 413±42 and 401±34 nmol/g for GM2 and GM3, respectively in untreated *Npc2^−/−^* mice; mean ± SD), and a significant reduction was still sustained in a 272-day old mouse.

Comparison of age-matched CD-treated and untreated *Npc2^−/−^* mice revealed less CD68 labeling in brains of treated animals, suggesting a decrease in neuro-inflammation. Evaluation of end-stage untreated *Npc2^−/−^* mice and age-matched CD-treated *Npc2^−/−^* mice also revealed more surviving Purkinje cells in the cerebellum of treated animals, although all remaining Purkinje cells still exhibited cholesterol accumulation (data not shown). The aforementioned results are analogous to those found in chronically treated *Npc1^−/−^* mice and demonstrate that *Npc2^−/−^* mice also benefit from treatment with CD.

### Effect of CD treatment on free sphingosine levels in NPC mice

**Figure 7:**
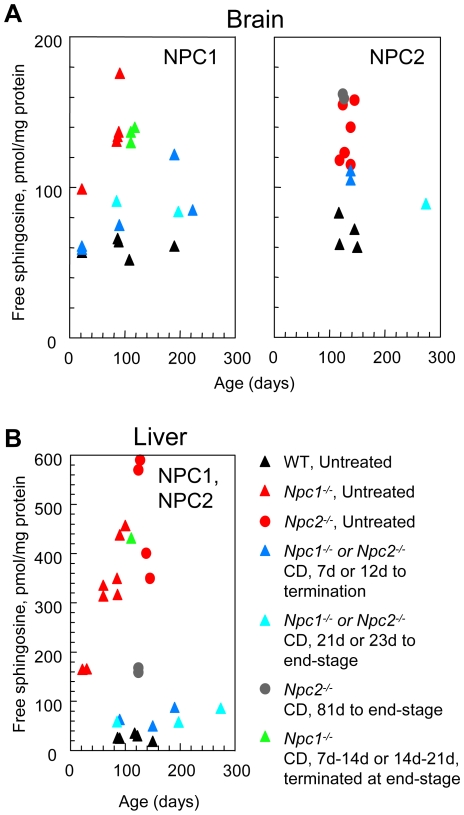
Free sphingosine concentrations in NPC disease following CD treatment. (A) Biochemical analysis of sphingosine in brain of untreated and CD-treated *Npc1^−/−^* and WT mice revealed that chronic CD treatment reduced sphingosine concentrations in affected mice, even when treatment was initiated post-weaning. *Npc2^−/−^* mice exhibited a similar trend, although the effect was less pronounced. (B) Biochemical analysis of liver from untreated and CD-treated *Npc1^−/−^* and *Npc2^−/−^* mice showed that with chronic CD treatment, sphingosine accumulation was reduced to levels near those found in WT.

In normal tissues free sphingoid bases (essentially sphingosine and sphinganine) are only present in minute amounts, similar to GM2 and GM3 gangliosides in the brain. In NPC patients and animal models, sequestration of free sphingosine in lysosomes contributes to the multiple lipid storage pattern, with a many-fold increase in liver, spleen and fibroblasts, but only a modest increase in brain [11], [24]–[25]. Free sphingosine is thus another interesting biomarker of NPC and, due to its free amino group, a putative offending metabolite. Indeed, Platt and colleagues [26] recently postulated that this compound might constitute a major factor of cell dysfunction in NPC. It was therefore also studied in the brains and selected livers of CD-treated and untreated mice. As shown in Fig. 7A, free sphingosine levels, already high in the brain of 22-day old *Npc1^−/−^* mice, increased further to reach a range of 130–180 pmol/mg protein, compared to 60±6 pmol/mg protein in WT mice (mean ± SD). Very similar levels were found in brains of *Npc2^−/−^* mice (Fig. 7A). Early chronic CD treatment normalized the free sphingosine level in brain of the 22-day old *Npc1^−/−^* mice, and significantly reduced values were still observed in mice treated from the early post-weaning period. A similar (although less pronounced) trend was found for *Npc2^−/−^* mice. The effect of an early one-week treatment (P7–P14) was, however, not sustained in late stage *Npc1^−/−^* mice, and chronic treatment starting at P81 was inefficient in *Npc2^−/−^* mouse brains. Livers from *Npc1^−/−^* and *Npc2^−/−^* mice showed 10–20 fold increased levels of free sphingosine (Fig. 7B), as well as of free sphinganine (data not shown), in good accordance to previously published data [24]. All chronic treatment regimens, even with a late start, appeared efficient in reducing free sphingosine accumulation in liver (Fig. 7B). By contrast, one single week of early treatment from P7 to P14 or P14 to P21 did not result in decreased levels in an end-stage mouse (Fig. 7B), a result consistent with the massive storage of cholesterol and sphingomyelin observed in the livers of those mice (Fig. S4).

### Chronic treatment of MPS IIIA and GM1 gangliosidosis mice with CD does not improve disease state

In addition to NPC disease, several other lysosomal diseases are characterized by accumulation of cholesterol and GSLs [27]. We hypothesized that these diseases might likewise benefit from treatment with CD. Mucopolysaccharidosis type IIIA (MPS IIIA) is caused by a deficiency in sulfamidase, a lysosomal enzyme necessary for the catabolism of the glycosaminoglycan, heparan sulfate [28]. In addition to heparan sulfate storage, neurons are known to accumulate GM2 and GM3 gangliosides and cholesterol [29]. GM1 gangliosidosis is also the result of a lysosomal enzymatic deficiency, β-galactosidase, which catabolizes GM1 to GM2 ganglioside, making GM1 the primary storage material. Cholesterol accumulation also occurs secondarily to that of GM1 in these mice [30] which, however, do not accumulate significant amounts of GM2 and GM3.

**Figure 8:**
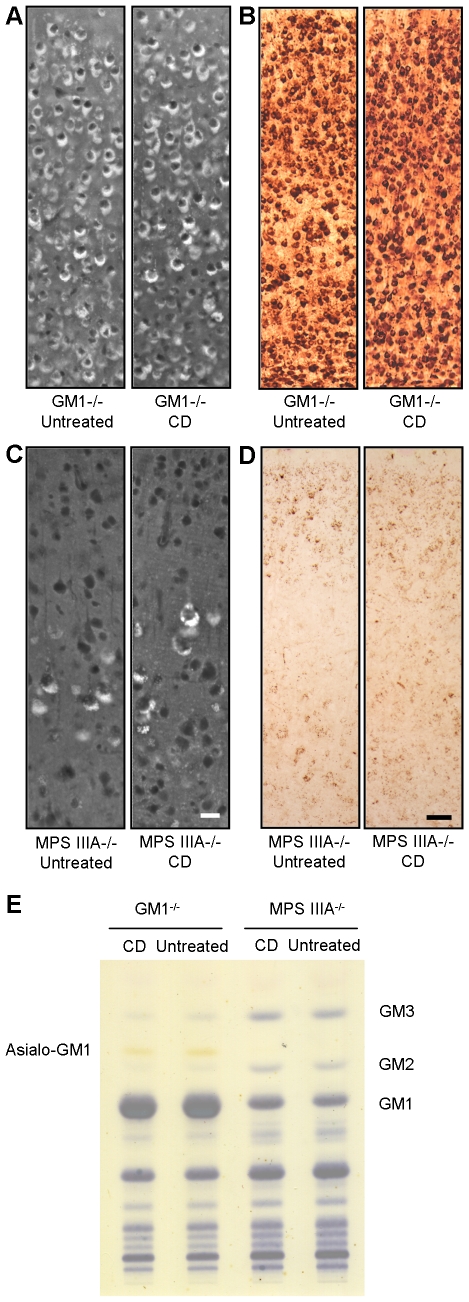
Chronic CD treatment study in GM1 and MPS IIIA mice. (A, B) Filipin labeling of unesterified cholesterol (A) and IHC of GM1 ganglioside (B) in neocortex of untreated (138 days old) and CD-treated (start at P21; 138 days old) mice with GM1 gangliosidosis. Brown punctae indicate GM1 accumulation within cortical neurons. No differences were observed in either filipin or GM1 labeling between CD-treated and untreated GM1 mice. (C, D) Filipin labeling of unesterified cholesterol (C) and IHC of GM2 ganglioside (D) in neocortex of untreated (140 days old) and CD-treated (start at P30; 140 days old) MPS IIIA mice. As with GM1 mice, no differences were observed between CD-treated and untreated MPS IIIA mice. (E) No reductions in GM1, Asialo-GM1, GM2, or GM3 ganglioside levels were seen in cerebral homogenates of CD-treated GM1 and MPS IIIA mice compared to untreated mice as evidenced by the thin-layer chromatography plate. Images taken at 20X (A, C) and 10X (B, D); scale bars 20 µm (C, also applicable to A) and 50 µm (D, also applicable to B).

Mice with MPS IIIA and GM1 diseases were treated with CD using protocols similar to the NPC studies. Since onset of clinically-evident brain dysfunction is documented to occur later in life for both mouse models (>5 mos) [31]–[33], analysis was limited to changes in brain storage of cholesterol and gangliosides. Short term studies in the MPS IIIA mice (analogous to the two week study carried out in the NPC1 mice) did not reveal detectable changes in either cholesterol or ganglioside (data not shown). Longer duration, chronic injections of CD also showed no evidence of impact on storage, even when initiated shortly after weaning (P21 or P30) and continued for over 3 months in both models (Fig. 8A–E).

Overall, treatment of both MPS IIIA and GM1 mice with CD showed no detectable benefit in reduction of either cholesterol or GSL storage. These results differed substantially from the ameliorating effects of CD treatment in NPC mice, which included a significant reduction of both cholesterol and GSL accumulation in treated *Npc1^−/−^* and *Npc2^−/−^* mice.

## Discussion

The use of combination therapy consisting of *N*B-DNJ and allopregnanolone in *Npc1^−/−^* mice had a synergistic effect in ameliorating disease progression beyond that seen with either monotherapy. Additionally, we found that treatment with CD, the vehicle for allopregnanolone, also provided benefit. As a result, we re-examined the proposed beneficial effect of allopregnanolone by utilizing different vehicles or a lower concentration of CD. These studies showed no significant differences in onset of clinical disease, in longevity, or in storage of cholesterol and GSLs between *Npc1^−/−^* mice receiving allopregnanolone in DMSO or corn oil compared to vehicle-only controls. Furthermore, while *Npc1^−/−^* mice treated weekly with allopregnanolone/5% CD or 5% CD alone did show an increase in lifespan beyond that seen in untreated *Npc1^−/−^* mice, the two groups did not differ significantly when compared with each other. Overall these data indicate that administration of CD (even at low concentrations), has a greater impact on ameliorating disease progression in NPC1 mice than does the administration of allopregnanolone without CD. These findings are further supported by recently published work in which a single injection of allopregnanolone in CD given to *Npc1^−/−^* mice did not increase lifespan beyond those mice receiving only the vehicle, CD [19].

Results from the above studies led us to investigate further the role of CD in ameliorating disease in treated *Npc1^−/−^* mice. Remarkably, *Npc1^−/−^* mice receiving CD at P7 and then every other day for two weeks revealed little to no intraneuronal accumulation of either cholesterol or gangliosides. Biochemical analysis also showed that these treated mice exhibited ganglioside and sphingosine levels comparable to WT controls. Furthermore, ultrastructural examination of neurons in treated mice revealed few to no PCBs and no other detectable morphological alterations. This apparent normalization of most CNS neurons was also accompanied by expression of the autophagosome marker, LC3-II, at a level resembling WT, while untreated *Npc1^−/−^* animals exhibited a nearly two-fold increase.

Studies examining chronic administration of CD initiated at P7 were found to provide the most significant impact on NPC disease progression. Treated mice showed delayed onset of ataxic gait and tremor, significantly increased lifespan, and greatly reduced accumulation of cholesterol, GSLs, and sphingosine. Treatment with CD also led to the rescue of some cerebellar Purkinje cells outside of lobule X as well as a decrease in a marker of neuro-inflammation, CD68, in the brains of treated *Npc1^−/−^* mice. LC3-II levels in the cerebellum of chronically treated *Npc1^−/−^* mice were again decreased when compared to untreated controls and very similar to WT levels, analogous to results of the short term study. Importantly, *Npc1^−/−^* mice administered CD beginning post-weaning (P21–P25) instead of P7 also demonstrated similar beneficial effects. Finally, comparison of CD-treated *Npc1^−/−^* mice using different routes of administration suggested that SC injections were slightly more effective in ameliorating NPC1 disease progression than IP administration. Published research has shown conflicting results in terms of the benefit of CD in amelioration of NPC1 disease [16], [19], [34]. However, our current results indicate that chronic, every other day treatment with CD, even when initiated post-weaning, proved significantly more beneficial than single [16], [19] or weekly injections of CD began at P7 and is the most efficacious of any compound tested to date in delaying NPC disease.

Confocal analysis of cerebral cortex from chronically-treated *Npc1^−/−^* mice revealed that many neurons were essentially devoid of cholesterol storage whereas neighboring cells sometimes exhibited significant accumulation. This finding indicates that CD may impact brain cells differently depending on their type. Interestingly, while some neurons without cholesterol storage also lacked GSL storage, many others displayed numerous GM2 and/or GM3-labeled storage bodies. Cholesterol-sequestering cortical neurons without accompanying ganglioside storage were not observed. What these observations mean in terms of a CD mechanism, or the function of NPC1 and NPC2 proteins, is not presently known. However, the finding is remarkably similar to what was observed in studies in which complex ganglioside synthesis was genetically blocked in NPC1 mice [11], [12]. Here, lack of gangliosides other than GM3 and GD3 led to dramatically reduced cholesterol storage in cerebral cortex and other brain areas. Cortical neurons that did persist with cholesterol storage were found to always accumulate GM3 ganglioside, whereas those neurons without GM3 lacked evidence of cholesterol sequestration [12]. While substantial evidence [8], [9] supports a direct role for the NPC1 and NPC2 proteins in cholesterol homeostasis, the persistence of ganglioside storage in chronically CD-treated NPC mice, and the apparent dependence of cholesterol sequestration on GM3 in the studies mentioned, continues to raise questions about the overall role of gangliosides in NPC disease in relation to cholesterol sequestration.

Treatment with CD in the NPC1 mouse model has shown significant beneficial effects on CNS neurons, yet the mechanism through which these effects are facilitated is unknown. One important but unresolved issue involves the permeability of the blood brain barrier (BBB) to CD. CD is a large molecule (molecular weight≈1396 Da) and there are contradictory findings in terms of its penetration of the BBB. *In vivo* studies in which mice were injected intravenously with radiolabeled CD and terminated 1 hour later have suggested that CD does not cross the BBB [34]. Yet a mechanism by which CD could have such striking effects on brain cholesterol and GSL storage in NPC disease without gaining direct access to neurons is difficult to envision. Conceivably, CD may complex with circulating 24S hydroxycholesterol (24S) or other sterols in the bloodstream and in turn create a “sink” which might have the ability to enhance cholesterol egress from brain. Such a mechanism would suggest a feedback loop in which cholesterol homeostasis within the brain could be influenced by levels of 24S or other circulating sterols within the bloodstream, however, no such mechanism is presently known.

Experiments examining cyclodextrins with regard to permeability using an *in vitro* model of the BBB have indicated that a small percentage of CD may be transported across the barrier [35]. Furthermore, there is evidence that use of cyclodextrins as vehicles for pharmacologic agents significantly facilitates their entry into brain [16], [36]. If CD does enter the brain, it presumably could exert its impact on cholesterol and GSL accumulation by acting at the plasmalemma of brain cells or after being internalized. One scenario, recently suggested by Dietschy and colleagues, places CD directly in the E/L system of neurons in *Npc1^−/−^* mice where it may act as a substitute for the defective NPC1 protein [19]. This idea implies that after crossing the BBB, HCD is endocytosed by neurons, complexes with cholesterol in LEs/LYs and together with the NPC2 protein facilitates movement of stored cholesterol. However, our studies also indicate that CD treatment is essentially of equal benefit to NPC2-deficient mice. This suggests that CD can replace the NPC1 protein, the NPC2 protein, or an entire cholesterol shuttling mechanism believed controlled conjointly by the two NPC proteins [9]. Perhaps more likely, if CD reaches the E/L system, it can complex with compounds other than cholesterol, for example, BMP or other phospholipids [37] and thereby change E/L membrane dynamics and cholesterol flux. It has been shown, for example, that BMP controls the cholesterol storage capacity of late endosomes [38]. CD may also have the ability to modify the internal environment of the LE/LY compartment in other ways, for example, by altering pH [39], and in some manner facilitate cholesterol flux and/or GSL catabolism. Any of these mechanisms conceivably could lead to normalized trafficking and clearance of accumulated cholesterol and GSLs, but all require CD to enter the neuronal E/L system.

Another scenario to explain the remarkable effect of CD on cholesterol and GSL storage in neurons, but not requiring direct access to the E/L system, would be an interaction with cholesterol at the neuronal plasmalemma. CD is known to have the ability to extract and deplete cholesterol from the cell membranes [23], [37], the result of which could be a redistribution of cholesterol from the E/L system to the plasmalemma. This would be consistent with the view that cholesterol accumulation in NPC cells represents a dynamic and mobile storage pool [40], with presumably, redistribution of stored cholesterol allowing secondarily for proper processing of GSLs. Our finding that CD is beneficial in reducing cholesterol and GSLs in neurons lacking either NPC1 or NPC2 proteins, but not in neurons in GM1 gangliosidosis or MPS IIIA, may be revealing of the importance of cholesterol's mobility in the NPC-affected cell compared with other lysosomal diseases.

A third potential mechanism of action for CD might involve a partial or modified BBB penetration. *In vitro* studies examining the effects of cyclodextrins on cholesterol removal from macrophage foam cells have shown that at low concentrations, CD can act as a shuttle to catalyze the flux of cholesterol between cell membranes and serum lipoproteins and at high concentrations, it can act as a sink for cholesterol [41]. Potentially, CD could simply complex with brain interstitial cholesterol and facilitate its transport into the circulation. CD could also extract cholesterol from the plasmalemma of endothelial cells lining the BBB and either shuttle cholesterol to acceptor molecules or act as a sink within the bloodstream as discussed earlier. Alternately, a scenario in which CD penetrates the BBB but does not enter the neuron could involve entry into vascular endothelial cells where its effects are exerted from within these frontline BBB cells, or transcytosis across these cells followed by endocytosis into adjacent astrocytes. Astrocytes are known to provide cholesterol to neurons and are poised to exert significant control over cholesterol homeostasis in neuronal cells [42]–[43]. Conceivably, normalization of cholesterol metabolism within diseased astrocytes could lead to an indirect normalization of cholesterol pools within neurons. Correction of the metabolic defect in astrocytes may then allow neurons to overcome their own metabolic defect, in turn leading to enhanced processing and trafficking of both cholesterol and GSLs. Consistent with this scenario, a recent study in which functional NPC1 protein was expressed in an astrocyte-specific manner in *Npc1^−/−^* mice was reported to lead to increased longevity and decreased neuronal storage of cholesterol [44].

CD is approved for use as a vehicle for drug delivery by several different routes of administration, including parenteral, oral, dermal, and transmucosal [45]. Toxicology studies in animal models, as well as metabolism and pharmacokinetics studies in humans, have shown CD to be well tolerated and any histopathological changes to be reversible [46]. Cyclodextrins are degraded by α-amylases, but those with substituents on the hydroxyl groups, such as CD, are more resistant to enzymatic degradation [23]. Futhermore, when administered parenterally, β-cyclodextrins are excreted almost completely intact in urine. It is possible that after complexing with cholesterol, CD travels through the bloodstream to the kidneys, where it is excreted in urine still complexed with cholesterol. However there were no noticeable differences in cholesterol accumulation within the kidneys of chronically-treated versus untreated *Npc1^−/−^* mice. Examination of cholesterol storage in livers of treated *Npc1^−/−^* mice revealed a shift from hepatocytes, which exhibit remarkable accumulation in untreated *Npc1^−/−^* mice, to apparent Kupffer cells in the livers of CD-treated mice. The cholesterol accumulation in Kupffer cells of liver from treated *Npc1^−/−^* mice was also noted in liver from treated WT mice. Indeed, the livers of CD treated *Npc1^−/−^* and WT mice looked remarkably similar in terms of cholesterol distribution. Analysis of lungs from untreated and CD-treated *Npc1^−/−^* mice revealed that cellular cholesterol storage was present in both groups. However, lungs from a long-term chronically treated *Npc1^−/−^* mouse at end-stage (approximately 14 weeks older than the *Npc1^−/−^* mice mentioned above) had a consolidated appearance and showed substantial cellular cholesterol accumulation, raising concerns about possible pulmonary complications with long-term CD treatment. These studies, as a whole, suggest that the effects of CD may differ by cell type, for example, when comparing neurons with hepatocytes or macrophage-lineage cells, and that multiple mechanisms of cholesterol mobilization may be involved.

Although the means by which CD exerts its beneficial effects in NPC disease are not understood, the outcome of CD treatment is clearly remarkable. It leads to delay in onset of clinical signs, a significant increase in lifespan, a reduction in cholesterol and ganglioside accumulation in neurons, reduced neurodegeneration, and normalization of markers for both autophagy and neuro-inflammation. Understanding the mechanism of action for CD will not only provide key insights into the cholesterol and GSL dysregulatory events in NPC disease and related disorders, but may also lead to a better understanding of homeostatic regulation of these molecules within normal neurons. Furthermore, elucidating the role of CD in amelioration of NPC disease will likely assist in development of new therapeutic options for this and other fatal lysosomal disorders.

## Methods

### Animals and drug administration

*Npc1^−/−^* mice, along with WT littermates, were generated by crossing *Npc1^+/−^* males and females in-house. The NPC1 mouse (BALBc/*NPC* ^nih^) was originally obtained from Peter Penchev at the National Institutes of Health (Bethesda, MD). Mouse pups were gentoyped according to published protocols [47] and WT and *Npc1^−/−^* mice were enrolled in the combination treatment study. Starting at P7 and weekly thereafter, some mice were injected SC with either 20% CD (control; 4000 mg/kg; H107, Sigma Aldrich, St. Louis, MO) or allopregnanolone (dissolved in 20% CD; 25 mg/kg; P3800-000, Steraloids, Newport, RI). Additionally, some mice were injected IP every day starting at P10 until P23 with either saline (control; 0.9% normal saline; 104 6816, Fisher Scientific, Waltham, MA) or miglustat (dissolved in saline; 300 mg/kg; a gift from Oxford GlycoSciences, Abingdon, UK/Celltech UK, Slough, Berkshire, UK). Following weaning at P23, miglustat and combination treated mice were housed individually in cages and fed powdered chow (Lab Diet 5058, PMI Nutrition International, LLC, Brentwood, MO) to which miglustat was added daily (1200 mg/kg) [14]. *Npc1^−/−^* mice, along with WT controls, were terminated when mice had at least two of the three signs considered to be end-stage disease. Clinical signs of end-stage disease included hunched posture and reluctance to move about the cage, inability to remain upright when moving forward, and weight loss greater than 30% of peak weight (mean end-stage weight = 14.3 g). *Npc1^−/−^* and WT mice placed in the CD treatment studies were administered SC or IP injections of 20% CD (4000 mg/kg) beginning at either P7 or shortly after weaning (P21–P25). Injections were continued every other day until sacrifice. *Npc2^−/−^*, MPS IIIA, and GM1 mice, along with WT littermates, were generated by crossing heterozygotes from in-house colonies of each disease model. Like NPC1 mice, the NPC2 mouse model is also on a uniform background [BALB/c; 10]. The MPSIIIA model is of mixed genetic background, consisting mainly of C57BL/6 with contributions from 129SvJ, CD1, and SJL mouse strains [32]. The GM1 colony also has a mixed genetic background with the major contributing strain being C57BL/6 [31]. These animals were treated with CD using the same protocol as for *Npc1^−/−^* mice, the only variation being the date of first injection, which ranged from P7 to P30.

Treated *Npc1^−/−^* and *Npc2^−/−^* mice, along with WT littermates, were sacrificed at end-stage or with age-matched untreated controls for comparison. MPS IIIA and GM1 mice plus WT littermates were sacrificed after approximately 3 months of treatment with CD, along with untreated controls. Mice were deeply anesthetized with an IP injection of sodium pentobarbital (150 mg/kg) and when insensate, were transcardially perfused with 0.9% saline solution. Following perfusion, a craniotomy was performed and the right cerebrum and right half of cerebellum were removed, along with liver and kidney, which were immediately frozen at −80°C for biochemical analyses. Mice were re-perfused with 4% paraformaldehyde in 0.1M phosphate buffer (PB) and additional tissues were collected (remaining half of brain, liver, kidney, spleen, and lung) and immersion fixed overnight in 4% paraformaldehyde/PB. Tissues were rinsed the following day and stored in PB at 4°C. All animal procedures were carried out according to guidelines approved by the Einstein College of Medicine Institutional Animal Care and Use Committee.

### Statistical analyses

JMP® software (JMP®, Version 7. SAS Institute Inc., Cary, NC) was used to analyze weight data from *Npc1^−/−^* and *Npc2^−/−^* mice in the treatment studies. There were no significant differences in weight between untreated and CD-treated WT mice, as Repeated Measures ANOVA revealed no main effect of treatment (F_treatment_ = 0.0027; p<0.8125) and no interactions between treatment and time (F_treatment×time_ = 0.4812; p<0.2041). There were also no significant differences between untreated and CD-treated WT weights when individual t-tests were performed at each time point. To determine if statistically significant differences between lifespan of treated and untreated groups occurred, a survival analysis using a Log-rank (Mantel-Cox) Test was performed in GraphPad Prism (GraphPad Software version 5.01, San Diego California USA).

### Antibodies and reagents

The following primary antibodies were purchased for use in immunohistochemistry and/or immunofluorescence: anti-GM3 ganglioside mAb (DH2, mouse IgG3, cell culture supernatant; 10-011; GlycoTech); anti-GM1 ganglioside pAb (IgG, serum; G2006-11; US Biological, Swampscott, MA); anti-CD68 mAb (rat IgG; MCA1957; AbD Serotec, Raleigh, NC); anti-calbindin mAb (mouse IgG, C9848) (Sigma-Aldrich, St. Louis, MO); and anti-LC3 pAb (rabbit IgG, NB100-2220; NOVUS Biologicals, Littleton, CO). Anti-GM2 ganglioside mAb (Mouse IgM, cell culture supernatant) was produced in-house from the 10–11 hybridoma line provided by Progenics Pharmaceuticals, Inc. (Tarrytown, NY). BC-theta was provided by Dr. Y. Ohno-Iwashita (Cellular Signaling Group, Tokyo Metropolitan Institute of Gerontology, Japan) (Iwamoto, 1997).

The following secondary antibodies and reagents were purchased for use in immunoperoxidase staining: biotinylated goat anti-mouse IgM (BA-2020), biotinylated goat anti-mouse IgG (BA-9200), biotinylated goat anti-rabbit IgG (BA-1000), Vectastain ABC kit (PK-4000), and DAB Peroxidase Substrate Kit (SK-4100) from Vector Laboratories (Burlingame, CA). The following secondary and tertiary antibodies were purchased for use in immunofluorescence: FITC-conjugated goat anti-mouse IgG (γ specific-Fc, 55517) from MP Biomedicals (Solon, OH); Alexa Fluor 633-conjugated goat anti-mouse IgM (μ chain, A21046), Alexa Fluor 546-conjugated streptavidin (S11225), and Alexa Fluor 488 Signal-Amplification Kit for FITC-conjugated probes (A11053) from Molecular Probes/Invitrogen (Carlsbad, CA). Filipin complex from *Streptomyces filipinensis* (F9765) was purchased from Sigma-Aldrich (St. Louis, MO). Peroxidase-labeled goat anti-rabbit IgG (PI-1000) was purchased from Vector Laboratories (Burlingame, CA) for use in western blotting.

### Immunohistochemical and filipin staining procedures

Immunoperoxidase staining was carried out according to previously published protocols [29]. Primary antibody dilutions were as follows: anti-GM2 (1∶5), anti-GM3 (1∶50), anti-GM1 (1∶5000), anti-calbindin (1∶3000), and anti-CD68 (1∶200). Secondary antibody dilutions were 1∶200 for biotinylated goat anti-mouse IgM, biotinylated goat anti-mouse IgG, and biotinylated goat anti-rabbit IgG.

Immunofluorescence was carried out according to previously published protocols [29]. Primary antibodies were diluted according to: anti-GM2 (1∶5), anti-GM3 (1∶5), and BC Theta (8 µg/ml). Secondary antibodies were diluted as follows: FITC-conjugated goat anti-mouse IgG (1∶200), Alexa Fluor 633-conjugated goat anti-mouse IgM (1∶300), Alexa Fluor 546-conjugated streptavidin (1∶750) and Alexa Fluor 488 Signal-Amplification Kit component A (1∶80). To visualize unesterified cholesterol, sections were incubated with filipin complex (0.005% dissolved in DMSO and diluted in PBS) or DMSO (control; diluted in PBS) and labeling was carried out according to previously cited protocols.

### Imaging procedures

Brightfield images were obtained using an upright Olympus AX70 microscope and MagnaFire camera. Images of filipin labeling were obtained on the same Olympus microscope but utilizing settings appropriate for acquisition of fluorescent images. Laser scanning confocal fluorescence images were obtained on a Zeiss 510 Duo V2 system using a 63X oil objective (NA = 1.4) and a zoom setting of 3. A multi-track mode, optimized for each fluorophore combination, was employed to help ensure no channel cross-talk. Digital images were further prepared for presentation using Metamorph software (Molecular Devices) and Adobe Photoshop.

### Electron microscopy

According to published protocols [32], fixed tissues were transferred to 0.1M cacodylate buffer, post-fixed in 2% glutaraldehyde, washed and post-fixed in osmium (1% osmium in 0.1% cacodylate buffer), dehydrated and embedded in Epon-aryldite. Ultrathin sections were cut from the plastic embedded blocks, stained with uranyl acetate and lead citrate, and examined with a Philips CM10 electron microscope.

### Biochemical lipid and protein analyses

Lipid analyses were carried out on frozen cerebral hemispheres and liver. Total lipid extracts were obtained as in Fujita et al. [48]. The procedures for ganglioside isolation and subsequent quantitation were as in previous studies [10], [49]. Analysis of free sphingoid bases was carried out by high-performance liquid chromatography as described by Rodriguez-Lafrasse et al. [25] using eicosasphinganin as an internal standard. Separation of o-phtalaldehyde derivatives was achieved on a 200×4.9 mm, 5 µm Spherisorb ODS2 column (Waters) and monitored by fluorometry.

Western blot analysis of LC3 was carried out according to the following method. Frozen brain tissue was homogenized in ice-cold lysis buffer (50 mM Tris-HCl, pH 7.5, 150 mM NaCl, 1% Igepal CA-630, 1% deoxycholic acid, 0.1% SDS supplemented with protease inhibitor cocktail), centrifuged (15000 rpm) for 30 minutes at 4°C and the supernatants (soluble fraction) were collected. Protein concentrations were determined using a BCA protein assay kit. For immunoblotting, samples were analyzed by SDS-PAGE (16% gels) under reducing conditions and transferred to Immuno-blot PVDF membranes. Membranes were blocked in 1x TBS, 0.1% Tween-20, 5% non-fat dry milk, 1% BSA, followed by incubation with an antibody to LC3 (2 µg/ml) and subsequent incubation with Peroxidase Labeled anti-Rabbit IgG secondary antibody (1∶5000). SuperSignal West Pico Chemiluminescent Substrate was used for protein detection on a KODAK 2000R imaging station. Protein quantification for each sample was performed by densitometric analysis using KODAK imaging software and normalized to actin in the same sample. This analysis was represented as LC3-II/Actin normalized to the wild-type untreated control.

## Supporting Information

Figure S1 Route of administration of CD in *Npc1^−/−^* mice. (A, B) Filipin labeling of unesterified cholesterol (A) and IHC of GM2 ganglioside (B) in neocortex of 22 day old *Npc1^−/−^* untreated (left panels) and CD-treated mice injected either SC (middle panels) or IP (right panels) starting at P7. Results indicated that while both routes of administration reduced cholesterol accumulation compared to untreated *Npc1^−/−^* mice, SC injections seemed to be more efficacious than IP injections. Images taken at 20X (A) and 10X (B); scale bars 20 µm (A) and 50 µm (B).
(4.05 MB TIF)

Figure S2 Cholesterol accumulation in visceral tissues from chronic CD treatment study in *Npc1^−/−^* mice. (A, B, C) Filipin labeling of unesterified cholesterol in kidney (A), liver (B), and lung (C) from untreated *Npc1^−/−^* (end-stage, 87 days old; first column), CD-treated *Npc1^−/−^* (age-matched, 90 days old; second column), CD-treated *Npc1^−/−^* (end-stage, 223 days old; third column),untreated (87 days old, fourth column) WT, and CD-treated WT (90 days old; fifth column) mice. No obvious differences were seen between cholesterol accumulation in kidneys of untreated versus CD-treated *Npc1^−/−^* mice (A). Filipin labeling indicated a shift of cholesterol storage from hepatocytes in untreated *Npc1^−/−^* mice to presumptive Kuppfer cells in CD-treated *Npc1^−/−^* mice, an observation also noted in CD-treated WT mice (B). Overall, filipin labeling of lung from both untreated and CD-treated *Npc1^−/−^* mice suggested the presence of more cholesterol accumulation than in lung from WT mice (C). Images taken at 20X; scale bar 20 µm (C, also applicable to A and B).
(4.65 MB TIF)

Figure S3 H&E staining of visceral tissues from chronic CD treatment study in *Npc1^−/−^* mice. (A, B, C) H&E staining of kidney (A), liver (B), and lung (C) from untreated and CD-treated *Npc1^−/−^* and WT mice (same mice as in Fig. S2). No obvious differences were noted in kidney between untreated and CD-treated mice (A). Staining of liver indicated the presence of lipid laden hepatocytes within untreated *Npc1^−/−^* tissue but these were not noted in any other mice (B). While macrophages were present in lung tissue from both untreated and CD-treated *Npc1^−/−^* mice, CD-treated mice showed increasing macrophage infiltration, especially as CD treatment continued to end-stage disease. Lipid-laden macrophages were not observed in either untreated or CD-treated WT tissue (C). Images taken at 20X; scale bar 20 µm (C, also applicable to A and B).
(8.44 MB TIF)

Figure S4 Effect of chronic CD treatment on lipid storage in liver of *Npc1^−/−^* mice. (A) Thin layer chromatographic profile of total lipids in liver tissue of *Npc1^−/−^* or WT mice, untreated (Unt'd) or CD-treated for the indicated period, visualized with the anisaldehyde reagent. Results indicate that chronic CD treatment, but not 1 week of early CD treatment (far right lane), normalizes lipid storage (Chol, GlcCer, BMP, and Sph) in *Npc1^−/−^* liver to levels near WT. The amount of lipid extract spotted corresponded to 2 mg wet tissue. Chol: unesterified cholesterol; GlcCer: glucosylceramide; BMP: bis(monoacylglycero)phosphate; Sph: sphingomyelin.
(1.34 MB TIF)
