# Supplementary figures and images for: Chronic Cyclodextrin Treatment of Murine Niemann-Pick C Disease Ameliorates Neuronal Cholesterol and Glycosphingolipid Storage and Disease Progression
Source: PLoS One. 2009 Sep 11;4(9):e6951. doi: 10.1371/journal.pone.0006951 (PMC2736622; doi:10.1371/journal.pone.0006951)

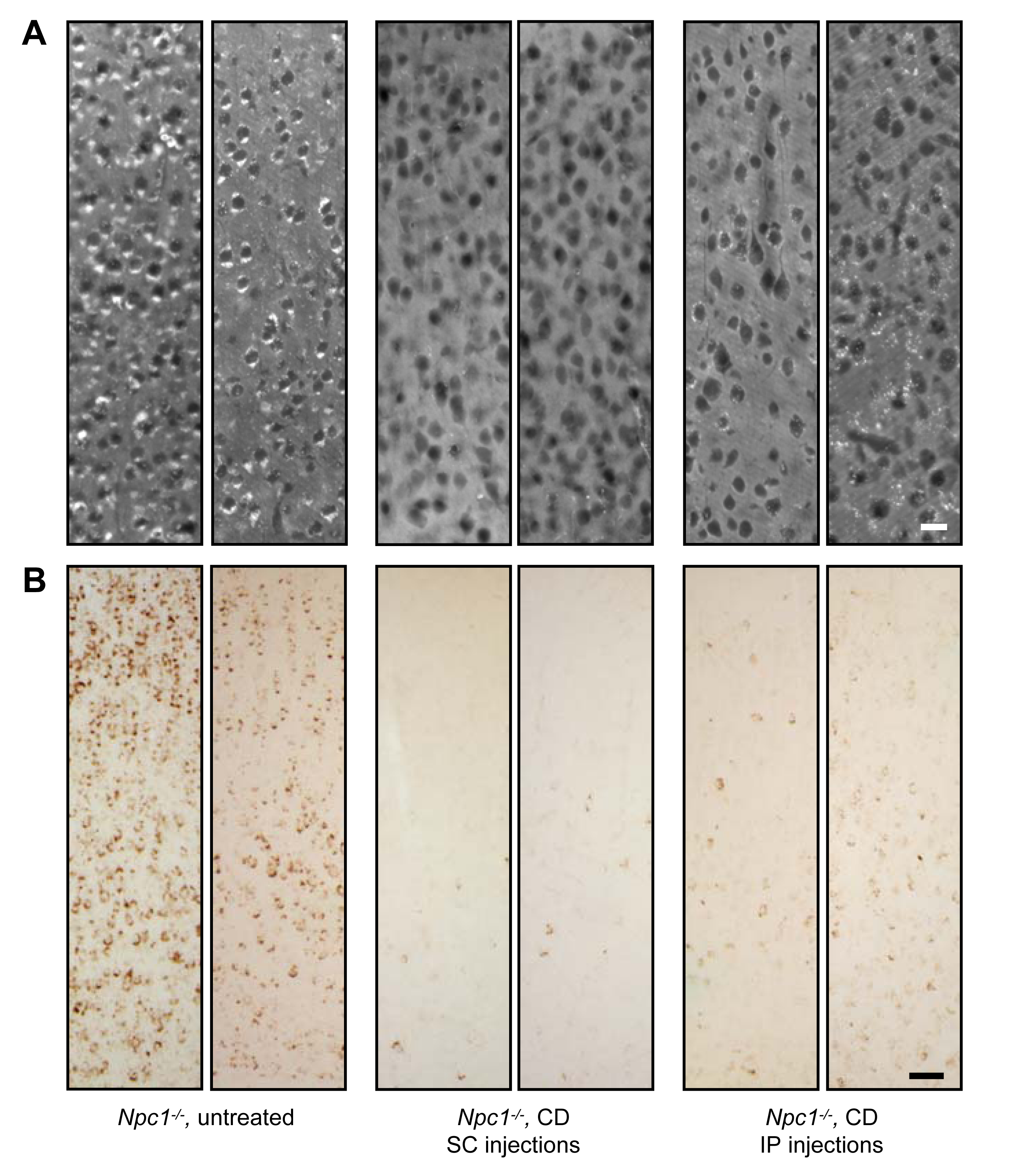

Supplement: Figure S1 — Route of administration of CD in Npc1−/− mice. (A, B) Filipin labeling of unesterified cholesterol (A) and IHC of GM2 ganglioside (B) in neocortex of 22 day old Npc1−/− untreated (left panels) and CD-treated mice injected either SC (middle panels) or IP (right panels) starting at P7. Results indicated that while both routes of administration reduced cholesterol accumulation compared to untreated Npc1−/− mice, SC injections seemed to be more efficacious than IP injections. Images taken at 20X (A) and 10X (B); scale bars 20 µm (A) and 50 µm (B). (4.05 MB TIF) [file pone.0006951.s001.tif]

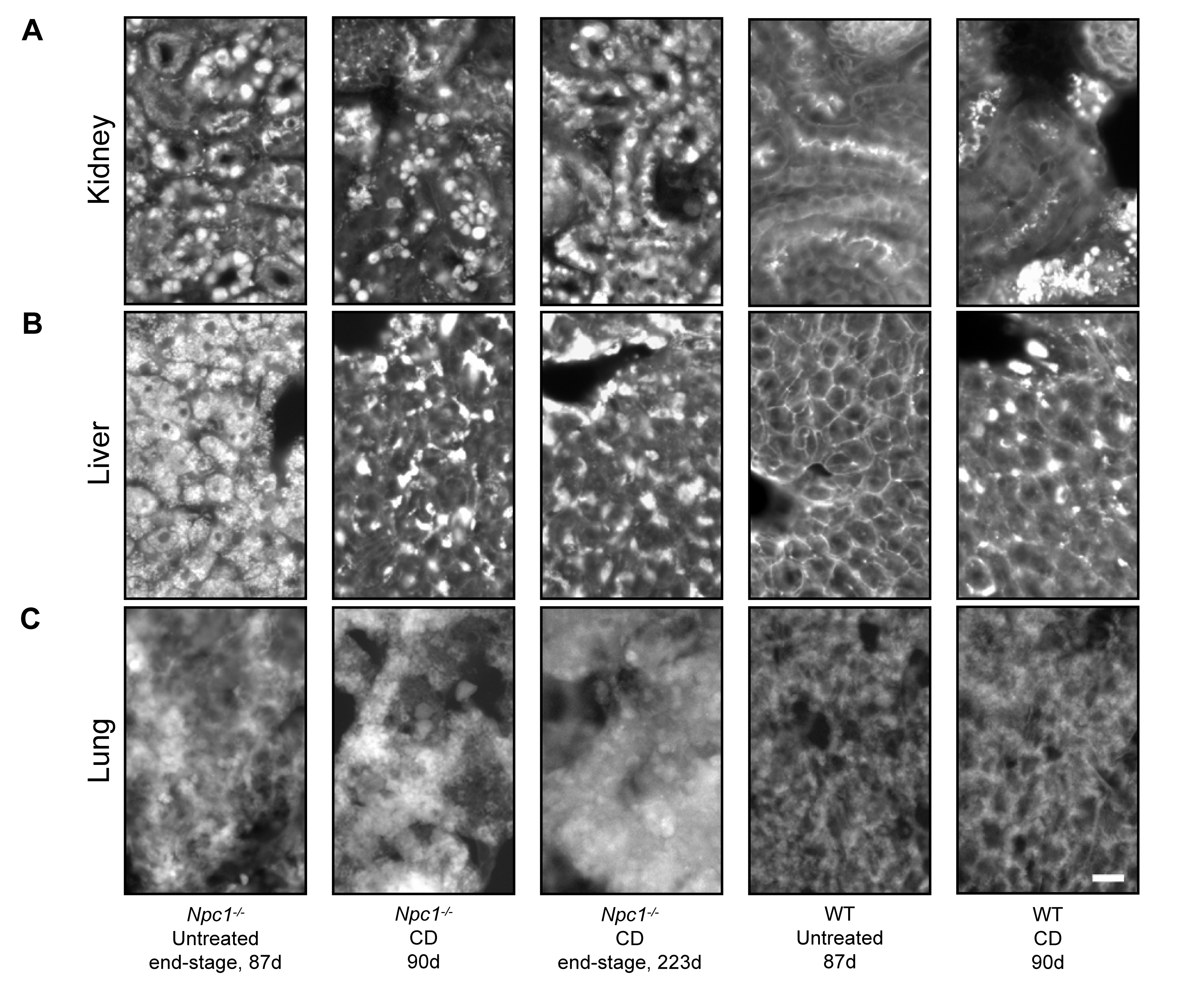

Supplement: Figure S2 — Cholesterol accumulation in visceral tissues from chronic CD treatment study in Npc1−/− mice. (A, B, C) Filipin labeling of unesterified cholesterol in kidney (A), liver (B), and lung (C) from untreated Npc1−/− (end-stage, 87 days old; first column), CD-treated Npc1−/− (age-matched, 90 days old; second column), CD-treated Npc1−/− (end-stage, 223 days old; third column),untreated (87 days old, fourth column) WT, and CD-treated WT (90 days old; fifth column) mice. No obvious differences were seen between cholesterol accumulation in kidneys of untreated versus CD-treated Npc1−/− mice (A). Filipin labeling indicated a shift of cholesterol storage from hepatocytes in untreated Npc1−/− mice to presumptive Kuppfer cells in CD-treated Npc1−/− mice, an observation also noted in CD-treated WT mice (B). Overall, filipin labeling of lung from both untreated and CD-treated Npc1−/− mice suggested the presence of more cholesterol accumulation than in lung from WT mice (C). Images taken at 20X; scale bar 20 µm (C, also applicable to A and B). (4.65 MB TIF) [file pone.0006951.s002.tif]

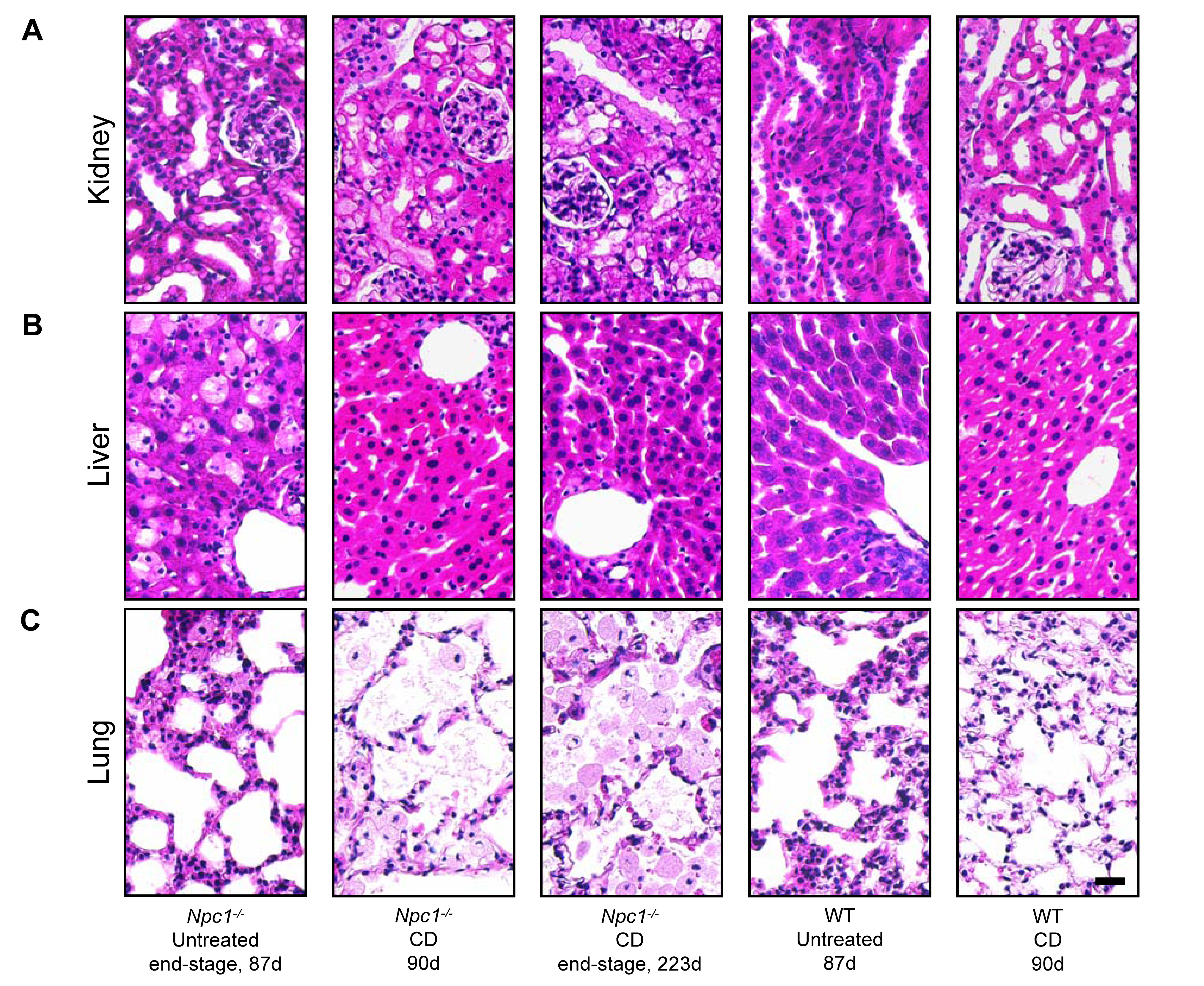

Supplement: Figure S3 — H&E staining of visceral tissues from chronic CD treatment study in Npc1−/− mice. (A, B, C) H&E staining of kidney (A), liver (B), and lung (C) from untreated and CD-treated Npc1−/− and WT mice (same mice as in Fig. S2). No obvious differences were noted in kidney between untreated and CD-treated mice (A). Staining of liver indicated the presence of lipid laden hepatocytes within untreated Npc1−/− tissue but these were not noted in any other mice (B). While macrophages were present in lung tissue from both untreated and CD-treated Npc1−/− mice, CD-treated mice showed increasing macrophage infiltration, especially as CD treatment continued to end-stage disease. Lipid-laden macrophages were not observed in either untreated or CD-treated WT tissue (C). Images taken at 20X; scale bar 20 µm (C, also applicable to A and B). (8.44 MB TIF) [file pone.0006951.s003.tif]

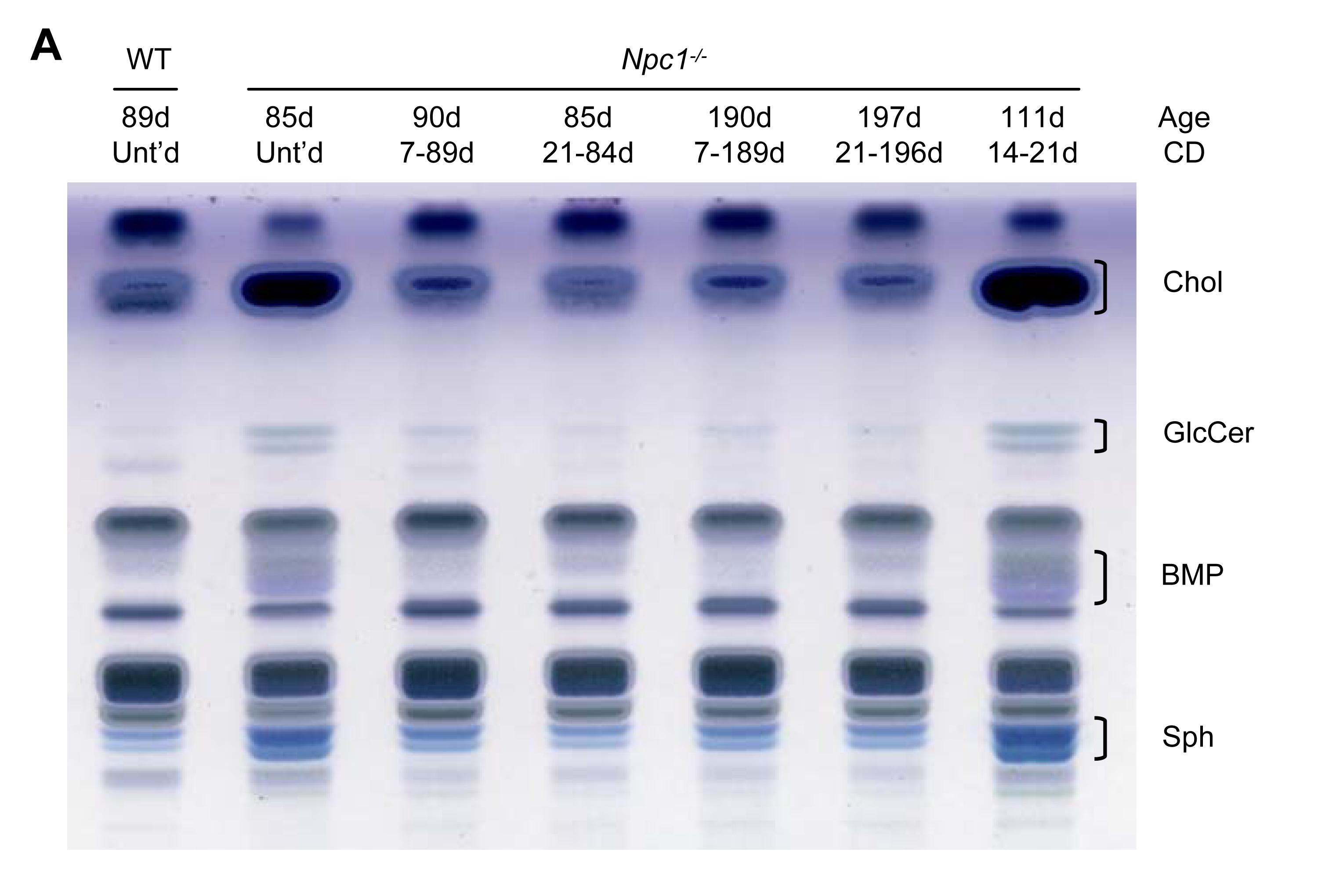

Supplement: Figure S4 — Effect of chronic CD treatment on lipid storage in liver of Npc1−/− mice. (A) Thin layer chromatographic profile of total lipids in liver tissue of Npc1−/− or WT mice, untreated (Unt'd) or CD-treated for the indicated period, visualized with the anisaldehyde reagent. Results indicate that chronic CD treatment, but not 1 week of early CD treatment (far right lane), normalizes lipid storage (Chol, GlcCer, BMP, and Sph) in Npc1−/− liver to levels near WT. The amount of lipid extract spotted corresponded to 2 mg wet tissue. Chol: unesterified cholesterol; GlcCer: glucosylceramide; BMP: bis(monoacylglycero)phosphate; Sph: sphingomyelin. (1.34 MB TIF) [file pone.0006951.s004.tif]
